# Critical review on the relationship between design variables and performance of dexterous hands: a quantitative analysis

**DOI:** 10.3389/fnbot.2024.1513458

**Published:** 2025-01-30

**Authors:** Lei Jiang, Chaojie Fu, Yanhong Liang, Yongbin Jin, Hongtao Wang

**Affiliations:** ^1^Center for X-Mechanics, Zhejiang University, Hangzhou, China; ^2^ZJU-Hangzhou Global Scientific and Technological Innovation Center, Zhejiang University, Hangzhou, China; ^3^State Key Laboratory of Fluid Power and Mechatronic System, Zhejiang University, Hangzhou, China; ^4^Institute of Applied Mechanics, Zhejiang University, Hangzhou, China

**Keywords:** dexterous hands, design variables, performance correlation, degrees of freedom, cross-correlation analysis

## Abstract

Dexterous hands play vital roles in tasks performed by humanoid robots. For the first time, we quantify the correlation between design variables and the performance of 65 dexterous hands using Cramér’s V. Comprehensive cross-correlation analysis quantitatively reveals how the performance, such as speed, weight, fingertip force, and compactness are related to the design variables including degrees of freedom (DOF), structural form, driving form, and transmission mode. This study shows how various design parameters are coupled inherently, leading to compromise in performance metrics. These findings provide a theoretical basis for the design of dexterous hands in various application scenarios and offer new insights for performance optimization.

## 1 Introduction

With the development of humanoid robots and dexterous hands, the application scenarios for dexterous hands have expanded significantly, covering manufacturing assembly lines (Xue and Yang, 2022; Tamada et al., 2013), medical care, and education (Li et al., 2004; Seguin et al., 2023). Each of these scenarios presents diverse performance requirements for dexterous hands. For instance, in medical and auxiliary robotics, flexibility and precise manipulation are crucial. In factory settings, the need for strong grasping force is more pronounced, while in complex work environments, speed and compactness are highly prioritized. The performance of dexterous hands is influenced by fundamental design variables, including the DOF, structural form, driving form, and transmission mode, among others.

In existing reviews on the performance of dexterous hands, Kashef et al. (2020) proposed evaluation criteria by comparing the performance of 28 linkage-driven dexterous hands, including force isotropy, shape adaptability (Yoon and Choi, 2017), and the number of phalanges. Belter et al. (2013) conducted a detailed analysis of the mechanical characteristics of 16 humanoid prosthetic hands in their review. Gama et al. (2014) assessed the technical characteristics of 27 dexterous hands, focusing on factors such as the number of fingers, DOF, actuator systems, and sensing processes. Kadalagere Sampath et al. (2023) reviewed the development history of dexterous hands and provided an analysis and brief overview of the performance of 12 dexterous hands. However, applying these evaluation criteria to practical designs still presents major challenges. Designers often struggle to achieve the desired performance by selecting appropriate design variables. Additionally, current research lacks a systematic quantitative analysis of the relationship between these design variables and performance metrics.

This study employs a statistical data-based method to quantify the correlation between different design variables and performance metrics through Cramér’s V. By analyzing the magnitude of the correlation values, we explore how different design variables impact the performance of dexterous hands. This provides a theoretical foundation for designing dexterous hands suitable for various application scenarios and addresses current research gaps. In contrast to the existing reviews on the performance of dexterous hands, our review is not limited to linkage-driven dexterous hands or prosthetic hands, but instead provides a comprehensive discussion on dexterous hands as a whole. Obtaining a large and comprehensive dataset of design variables and performance for dexterous hands is challenging. Nevertheless, our survey includes data on 65 dexterous hands, making it the most comprehensive review of dexterous hands. Furthermore, for the first time, we quantify the relationship between design variables and performance of dexterous hands using Cramér’s V, a novel approach in the field of review papers.

The structure of this paper is as follows: The second section outlines the data collection methods for dexterous hand design variables and performance metrics, and introduces the analysis method for determining the correlation between these design variables and performance metrics. The third section discusses the relationship between design variables and performance aspects such as speed, weight, fingertip force, and compactness. The fourth section provides a more in-depth analysis of design variables, including transmission mode, driving form, DOF, and structural form. The fifth section explores the coupling relationships between fingertip force and weight, and fingertip force and speed. The sixth section proposes a standardized framework for performance evaluation. Finally, the seventh section summarizes the main findings of this study and discusses future trends in the development of dexterous hand design.

## 2 Methods

This section outlines the methodology used to analyze dexterous hands. It describes how design variables and performance metrics are categorized and processed. We then analyze various methods for quantifying the correlation between these variables and metrics. Finally, we use Cramér’s V to assess the correlations between design and performance, ensuring transparency and consistency. Although the data is currently limited, the findings provide a solid foundation for evaluating and optimizing dexterous hand designs.

The design variables of a dexterous hand primarily include DOF, structural form, transmission mode, and driving form, while the performance metrics include speed, reliability, weight, fingertip force, compactness, workspace, stiffness, and robustness (Wassink et al., 2010; Dong et al., 2019). We uniformly process the design variables and performance metrics that are difficult to compare and analyze. The specific steps are as follows: 1. DOF statistics: The active DOF of the thumb is counted separately. 2. Speed standardization: Unified conversion to °/s. 3. Unified expression of force: Unified expression of fingertip force. 4. Compactness calculation: The length of the finger is divided by the thickness, and the length and thickness are estimated by the introduction or picture measurement in the paper.

Some of these design variables and performance metrics are continuous, while others are categorical, and certain metrics are difficult to quantify directly. To facilitate the analysis, we converted the continuous performance metrics—fingertip force, speed, weight, and compactness—into discrete categorical variables. Specifically, fingertip force was divided into two categories: <12 N and ≥12 N; speed was categorized as <200°/s and ≥200°/s; weight was split into <0.5 kg and ≥0.5 kg; and compactness was classified as <5.5 and ≥5.5. In the same way, the design variable DOF was divided into two groups: <3 and ≥3. The structural form was categorized based on the location of the motors, distinguishing between designs with built-in motors and those with external motors. The driving form was divided into two groups: fully actuated and underactuated. Transmission modes were classified into first-level and second-level categories. The first-level transmission mode refers to the transmission from the drive to the finger, including tendon-driven, gear-driven, ball screw-driven, and linkage-driven systems. The second-level transmission mode refers to the transmission within the finger itself, including tendon-driven, gear-driven, belt-driven, and linkage-driven systems. For ease of analysis, we consolidated the first-level and second-level transmission modes into two broader categories: tendon-driven and linkage-driven.

We reviewed various methods to quantify the correlation between design variables and performance metrics. Principal Component Analysis (PCA) is a linear dimensionality reduction technique that works effectively with continuous design variables. However, PCA is not directly applicable to categorical design variables, such as transmission mode and driving form, as these do not have an inherent numerical relationship. To handle categorical data, we considered Multiple Correspondence Analysis (MCA), a technique specifically designed for analyzing multi-category data. MCA can uncover hidden relationships between categorical variables and reduce the dimensionality of the data by mapping it to a lower-dimensional space. However, applying MCA to analyze the design variables and performance metrics of dexterous hands requires complete data for both design variables and performance metrics across all samples, which is challenging due to the limited dataset and the difficulty in identifying clear patterns. Another method, mutual information, quantifies the correlation between discrete variables based on information theory. Despite its utility, mutual information values are influenced by the base of the logarithm used, making it difficult to directly interpret the strength of the correlation. It is more effective in comparative scenarios. In contrast, Cramér’s V is particularly valuable for measuring the strength of association between two categorical variables. It is applicable to various types of categorical data and does not require assumptions like normality, making it suitable for handling non-normally distributed or complex categorical data. Furthermore, Cramér’s V not only identifies associations between variables but also quantifies their strength. Values between 0.40 and 0.70 indicate a moderate to strong relationship with statistically significant relevance, providing a clear method to compare correlation strengths. Given these advantages, we chose to use Cramér’s V to explore the relationship between design variables and performance metrics.

This study not only investigated the existing commercial dexterous hand, but also systematically searched electronic databases such as Google, Google Scholar, ScienceDirect, IEEE Xplore and Scopus, and investigated more than 370 articles.

When dealing with incomplete data, we take the following methods: 1. Data Completion: Speculation through similar designs in other literature or measurement based on diagrams. 2. Data exclusion: data points with more missing information and unreasonable speculation are excluded. 3. Information merging: Consolidate the repeated information of the same dexterous hand in different literatures to ensure data consistency and accuracy.

Given the challenges associated with collecting large-scale data, some of the data sources used in our study may have inherent limitations or discrepancies. While we have made every effort to ensure the accuracy of the data, a small margin of error may exist in a few parameters. We believe these minor discrepancies have little impact on the overall research methodology and conclusions. We hope that with future standardization and improvements in data quality, these limitations will be further reduced. After detailed screening and processing, the statistical data obtained are shown in Supplementary Table 1. Some dexterous hands ‘information is difficult to obtain, but according to some statistical information, it can still provide valuable analysis. These data are shown in Supplementary Table 2. Supplementary Tables 1 and 2 are provided at the supplementary materials. Through the above steps, the transparency and repeatability of the research are ensured as much as possible. However, due to the fact that the performance metrics of the dexterous hand has not yet formed a unified standard, the statistical data is still limited.

Based on the data of the above 65 dexterous hands, we use Cramér’s V to measure the correlation between design variables and performance metrics of dexterous hands. We constructed 20 contingency tables to show the joint frequency distributions between five design variables and four performance metrics. Supplementary Tables 3–22 are provided at the supplementary materials.

The expected frequency *Eij* for each cell in the table is calculated using the following formula:


$$E_{ij}=\frac{R_{i}C_{j}}{N}$$

where *Ri* is the sum of the *i*-th row, *Cj* is the sum of the *j*-th column, and *N* is the total sample size.

The chi-square statistic *χ*^2^ is then computed using the formula:


$$x^{2}=\sum \frac{{\left(O_{ij}-E_{ij}\right)}^{2}}{E_{ij}}$$

where *Oij* represents the observed frequency in the *i*-th row and *j*-th column of the contingency table.

Cramér’s V is calculated using the following formula:


$$V=\sqrt{\frac{x^{2}}{N\times \min\left(k-1,r-1\right)}}$$

where *k* is the number of rows and *r* is the number of columns.

The distribution of Cramér’s V values for the design variables and performance metrics is shown in Table 1.

**Table 1:** The distribution of Cramér’s V values for the design variables and performance metrics.

Design variables	Performance metrics	Cramér’s V
First-level transmission mode	Compactness	0.246
First-level transmission mode	Weight	0.365
First-level transmission mode	Fingertip force	0.168
First-level transmission mode	Speed	0.490
Second-level transmission mode	Compactness	0.174
Second-level transmission mode	Weight	0.078
Second-level transmission mode	Fingertip force	0.294
Second-level transmission mode	Speed	0.062
Structural form	Compactness	0.037
Structural form	Weight	0.276
Structural form	Fingertip force	0.077
Structural form	Speed	0.354
DOF	Compactness	0.128
DOF	Weight	0.587
DOF	Fingertip force	0.164
DOF	Speed	0.330
Driving form	Compactness	0.263
Driving form	Weight	0.440
Driving form	Fingertip force	0.152
Driving form	Speed	0.520

**Figure 1:**
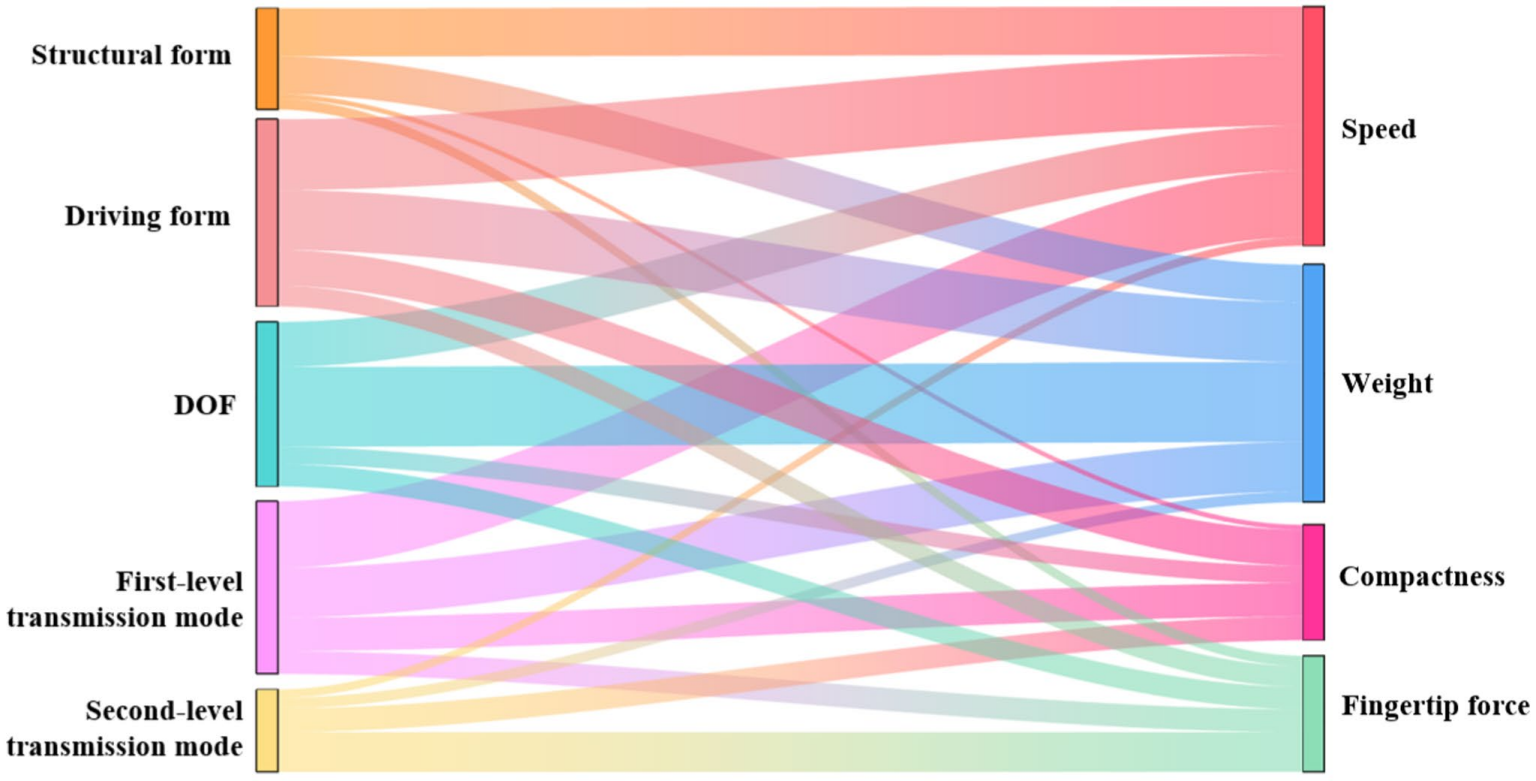
The Sankey diagram of the correlation value between the design variables and the performance of the dexterous hand.

The calculated Cramér’s V values were plotted to generate the following Sankey diagram, as shown in Figure 1. The left side represents the design variables of the dexterous hand, the right side represents the performance of the dexterous hand, and the thickness of the curve represents the correlation between the design variables and the performance. Then, the correlation value is analyzed. The higher the correlation value of the dexterous hand design variable is accumulated, the greater its influence in the whole system. Although the cumulative result of the correlation value is a composite indicator, it can provide a holistic perspective. The higher the cumulative correlation value of the performance of the dexterous hand, the stronger the correlation with the independent variable, and the easier the performance is optimized.

Cramér’s V values between 0.40 and 0.7 indicate a moderate to strong relationship with significant statistical relevance. The Sankey diagram reveals that the strongest correlation between design variables and performance is found between DOF and weight. Additionally, significant correlations are observed between the driving form and speed, the first-level transmission mode and speed, and the driving form and weight.

Although Cramér’s V can effectively evaluate the correlation between different dexterous hand design variables and performance, due to the limited amount of existing dexterous hand data, these correlation assessments may change with the increase of future data.

## 3 Design variables

This section provides a detailed analysis of key design variables for dexterous hands, with a focus on their structural aspects. It compares the different structural configurations associated with four critical design variables. The analysis examines common transmission modes, including linkage-driven and tendon-driven mechanisms, and highlights the characteristics of various structural forms within each mode. Additionally, it contrasts the effects of underactuated and fully actuated designs, specifying the corresponding structural configurations for each. The distribution of DOF in the human hand is also reviewed, along with an examination of how varying DOF affect performance. Special attention is given to the structural implementation of the key DOF—the base joint DOF in the finger. Finally, the paper compares built-in motor designs with external motor configurations, analyzing their respective impacts on performance.

### 3.1 Transmission mode

#### 3.1.1 Linkage-driven

The structure of dexterous hand based on linkage-driven mechanisms mainly includes two-bar linkages, ring four-bar linkages (R-FBL), cross four-bar linkages (C-FBL), five-bar linkages and multi-bar linkages. The two-bar linkages mechanism is simple in structure and light in weight, and is suitable for basic grasping and moving tasks. R-FBL and C-FBL provide more complex motion modes for high DOF operations; five-bar linkages and multi-bar linkages are suitable for delicate operation and complex tasks.

We have analyzed the natural motion ability (Khakpour and Birglen, 2013; Lee, 2010), the number of DOF and the use of series or parallel mechanisms of 21 kinds of dexterous hands based on linkage-driven mechanisms, as shown in Table 2. The results show that the DOF of the parallel mode is generally higher than that of the series mode, but it is still very difficult to achieve a dexterous hand with more than 3 DOF, mainly due to structural complexity and volume constraints. Nevertheless, the parallel mechanism has high bearing capacity, excellent motion accuracy and adaptability.

**Table 2:** There are 21 kinds of dexterous hands based on linkage-driven mechanisms.

	Structure scheme	Natural motion	DOF	Series/parallel
1 (Kim et al., 2021)	Two C-FBL, One R-FBL	Yes	3	Parallel
2 (Inspire Robots Dexterous Hand, n.d.)	Linkage transmission	No	2	Series
3 (Controzzi et al., 2016)	One C-FBL	No	2	Series
4 (Yan et al., 2017)	One FBL	No	3	Series
5 (Sensor Hand Speed, n.d.)	Linkage transmission	No	1	Series
6 (Yang et al., 2009)	One C-FBL, One R-FBL	Yes	2	Parallel
7 (Dechev et al., 2001)	Two C-FBL	Yes	1	Series
8 (Kyberd et al., 2001)	Two C-FBL	Yes	2	Series
9 (Cheng et al., 2017)	One coupled-adaptive multi-bar linkage, One C-FBL	Yes	2	Parallel
10 (Laliberté and Gosselin, 2001)	Two R-FBL	No	2	Series
11 (Stavenuiter et al., 2017)	Linkage transmission	No	2	Series
12 (Fukaya et al., 2000)	One multi-bar linkage, One C-FBL	Yes	2	Parallel
13 (Yoon et al., 2016)	Two five-bar linkages, One C-FBL	Yes	3	Parallel
14 (Zainul and Yamaura, 2012)	Two R-FBL equipped with a guiding slut	Yes	3	Parallel
15 (Wu et al., 2009)	Two C-FBL	Yes	3	Parallel
16 (Zhang et al., 2013)	One R-FBL, One C-FBL	Yes	2	Parallel
17 (Li et al., 2017)	One R-FBL, One C-FBL	Yes	2	Parallel
18 (Mu and Huang, 2007)	Parallelogram linkage	Yes	3	Parallel
19 (Gopura et al., 2017)	Two R-FBL	Yes	1	Series
20 (Khakpour and Birglen, 2013)	Six two-dimensional connecting rods	No	3	–
21 (Lee, 2010)	Two C-FBL	Yes	2	Series

We have counted the structural distribution of 21 different designs of dexterous hands based on linkage-driven mechanisms. Most of the linkage-driven dexterous hands use C-FBL and R-FBL. Among these mechanisms, the C-FBL and the R-FBL are easier to achieve natural motion and achieve better grasping performance.

#### 3.1.2 Tendon-driven

**Figure 2:**
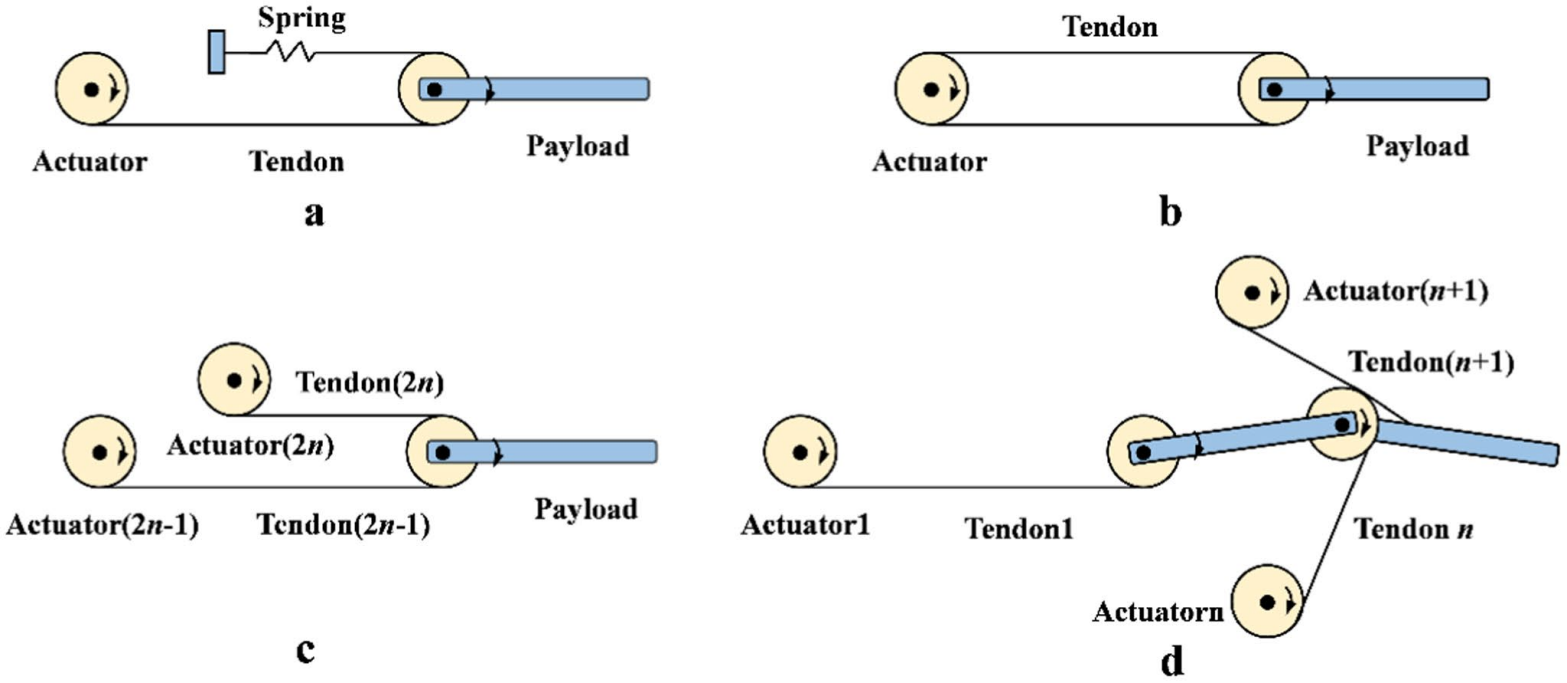
The structures of dexterous hands based on tendon-driven mechanisms. **(A)** N-type structure with N actuators and N tendons. **(B)** N-type structure with N actuators and 2N tendons. **(C)** 2N-type structure with 2N actuators and tendons. **(D)** N+1-type structure with N+1 actuators and tendons.

The structures of dexterous hand based on tendon-driven mechanisms mainly classified into N type, N + 1 type, and 2 N type (Pons et al., 1999). As shown in Figure 2, Figures 2A and 2B illustrate the N-type structure. In Figure 2A, the number of actuators is N, and the number of tendons is also N. The number of actuators is limited. In Figure 2B, the number of actuators is N, and the number of tendons is 2 N. Although the actuators are few, a pre-tightening mechanism is necessary to maintain tendon tension stability. In Figure 2C, the number of actuators is 2 N, and the number of tendons is also 2 N. This design has a large number of actuators, providing strong bearing capacity and excellent dynamic performance, but it also increases structural complexity and cost. In Figure 2D, the number of actuators is N + 1, and the number of tendons is also N + 1. Although the number of tendons is relatively small, each tendon needs to bear a large load. This design simplifies control and reduces actuator usage in certain applications.

In the dexterous hand based on tendon-driven mechanisms, structures with more than two active DOF typically utilize external motors. Tendon-driven dexterous hands can achieve four or even five DOF, a feat challenging to accomplish with linkage-driven mechanisms. Tendon-driven mechanisms not only enable high DOF motion but also adapt more effectively to complex operational requirements through the use of external motors and flexible tendon configurations. In contrast, the complex structures and fixed motion paths of linkage-driven mechanisms pose significant design and implementation challenges when increasing the DOF.

### 3.2 Driving form

Most dexterous hands use underactuated design to reduce the number of independent actuators, simplify control tasks (Dollar and Howe, 2011), and are flexible and adaptable to objects of irregular shapes and sizes. The thumb DIP joint is usually controlled separately, while the PIP and DIP joints of the other four fingers adopt a coupled underactuated mode to achieve natural grasping and manipulation (Kragten et al., 2011).

Common joint coupling methods include a single tendon or cable for each finger, fixed coupling (MCP, PIP, and DIP joints), linkage coupling, and compliant elastic springs. Single tendon or cable control is effective, but the structure is complex; the fixed coupling simplified control system has low flexibility; the coupling of the linkage is stable and suitable for complex motion. The compliant spring provides flexibility but has low control accuracy.

In fully actuated dexterous hands, each joint is equipped with an independent actuation system, enabling more sophisticated motion patterns. However, this increased functionality typically comes with a more complex structure, making maintenance and control more challenging.

### 3.3 DOF

According to Xiaohui (2022) hand pose estimation method, there are 26 DOF in the human hand, including 20 hand DOF and 6 wrist DOF. In the design of the dexterous hand, the DOF of the thumb is much larger than that of other fingers. At least 2 DOF are required to ensure that the dexterous hand can achieve basic grasping and manipulating behavior. In commonly used grasping and manipulating postures, the hand’s manipulation of objects is largely dependent on the thumb’s function. The function of the thumb accounts for 40% of the overall function of the hand (Faderani et al., 2023), and the grasping ability of the thumb accounts for more than 50% of the grasping ability of the hand (Deimel and Brock, 2016; Zhou et al., 2019).

The DOF of the fingers is crucial to the grasping and manipulating ability of the dexterous hand (Gopura et al., 2017). The high DOF allow the fingers to move and adjust their posture in more directions, enabling more complex grasping and manipulation. For example, multi-DOF fingers can more naturally mimic the movements of human hands, better adapting to objects of different shapes and sizes to achieve stable grasping. Although high DOF can improve flexibility, low DOF dexterous hands can achieve good grasping and manipulation performance in some cases. In addition, high DOF also increases design and control complexity, raising the cost and the risk of failure. Therefore, when designing a dexterous hand, it is necessary to strike a balance between the number of DOF and the system’s complexity to meet task requirements without introducing excessive complexity.

Each finger of the human hand can achieve lateral swing for grasping and manipulation in complex situations. The dexterous hand with low DOF often abandons the lateral swing DOF and only retains the DOF that bends into the palm, making it less anthropomorphic in performing many tasks. The lateral swing DOF (the base joint DOF in the finger) in existing dexterous hands is mainly realized through four methods. The first method uses a cross-axis linear mechanism, such as fisheye bearings, ball head buckles, or universal joints, to achieve two-directional rotation. This design offers a compact structure and a wide range of movement, but it is prone to wear and has lower precision. The second method adopts a non-crossing cross-axis linear arrangement, achieving two-directional rotation by keeping the axes parallel in the vertical direction compared with the first method, it is easy to manufacture, but the range of motion and flexibility are small. The third type adopts two sets of opposite bevel gears, and realizes the rotation in two directions by crossing the two axes in the vertical direction. The transmission efficiency is high and the precise motion control can be realized. The fourth method is to increase the pulley, increase the tension of the tendon-driven laterally, and realize the lateral tension of the tendon-driven by increasing the pulley. It is more complicated and harder to control.

### 3.4 Structural form

The built-in motors design (Odhner and Dollar, 2011) places motors directly inside the fingers or palm. This method can achieve a more compact structure, reducing the overall weight and volume of the hand. It also simplifies the transmission system, as it does not require long-distance transmission of torque and power. However, built-in motor designs also introduce heat dissipation problems and space constraints (Butterfass et al., 2001), which will limit the power and efficiency of the motors.

External motors are designed to place motors outside the arm or palm, with power transmitted to the fingers through a transmission mechanism. This design assists in heat dissipation, enabling the use of higher-power motors to improve hand performance (Hernando et al., 2023). External motors also make maintenance and replacement more convenient. However, the addition of the transmission system may increase the complexity and weight of the overall system, and could affect the flexibility and accuracy of the transmission chain. In design, the choice of built-in or external motors should be weighed according to the specific application requirements. Considerations include the flexibility requirements of the hand, space constraints, heat dissipation requirements (Nikafrooz and Leonessa, 2021), maintenance convenience, and motor power.

## 4 Performance metrics

This section examines the performance metrics of dexterous hands and their relationship with design variables. It covers four key metrics: speed, weight, fingertip force, and compactness. Each metric is first discussed in terms of its importance across various application scenarios. This is followed by an analysis of how the human hand performs in relation to the metric, along with a comparison to the performance of existing dexterous hands. A detailed exploration then examines the correlation between the design variables of dexterous hands and these performance metrics. Finally, recommendations are provided to improve the performance of each metric.

### 4.1 Speed

In some complex operations, the speed of the dexterous hand is crucial. For instance, when manipulating objects in the palm, the hand must quickly adjust its position and posture to meet changing needs. Chen Tao et al.’s manipulator object orientation adjustment experiment (Chen et al., 2023) and Huang et al.’s fast ball grasping experiment (Huang et al., 2023) show that the dexterous hand needs high speed and precision to work together to interact effectively.

The typical speed range of a human hand’s daily pick-and-place tasks is 172–200°/s (Biddiss and Chau, 2007). Tözeren suggested that the closure time of the dexterous hand should be 0.8 s to meet the requirements (Tözeren, 1999), while Detchev et al. suggested that a slower closure time of 1.0–1.5 s is sufficient to complete activities of daily living (Dechev et al., 2001). Therefore, a dexterous hand speed of around 200°/s can meet most daily application scenarios. However, the maximum bending speed of the human hand reaches 2,290°/s (Weir et al., 2009), which is difficult for a dexterous hand to achieve.

Currently, the maximum speed of dexterous hands typically does not match the highest speed of human fingers.

The driving form shows the strongest correlation with speed. Fully actuated dexterous hands are faster than underactuated ones because they enable independent control of each joint. In contrast, underactuated dexterous hands rely on passive compliance in their mechanical design, have longer transmission distances, and generally use less power.

The speed of the dexterous hand is second most strongly correlated with the first-level transmission mode, while the correlation with the second-level transmission mode is relatively weak.

**Figure 3:**
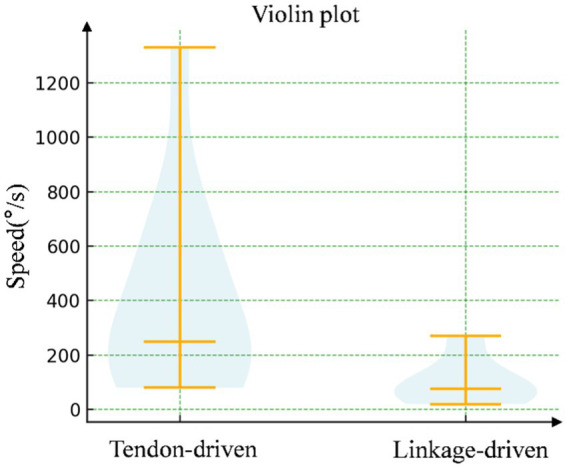
The first-level transmission mode and speed distribution of different dexterous hands.

The first-level transmission modes include linkage-driven, tendon-driven, ball screw-driven, and gear-driven. To standardize the variables, the speed distribution of dexterous hands based on linkage-driven and tendon-driven mechanisms is primarily analyzed. It can be seen from Figure 3 that the speed of the dexterous hand driven by the linkage is significantly lower than that of the dexterous hand driven by the tendon. This is because the tendon-driven mechanisms allow for long-distance transmission and the use of more powerful motors, making the tendon-driven dexterous hand generally faster.

The correlation between structural form and speed is weak. High-speed dexterous hands often use external motors, but some, such as the Inspire Robots Dexterous Hand, use built-in motors and achieve faster speeds by reducing the number of motors and optimizing motor performance through a reduced DOF. To achieve good overall performance, using external motors is often the more practical solution.

**Figure 4:**
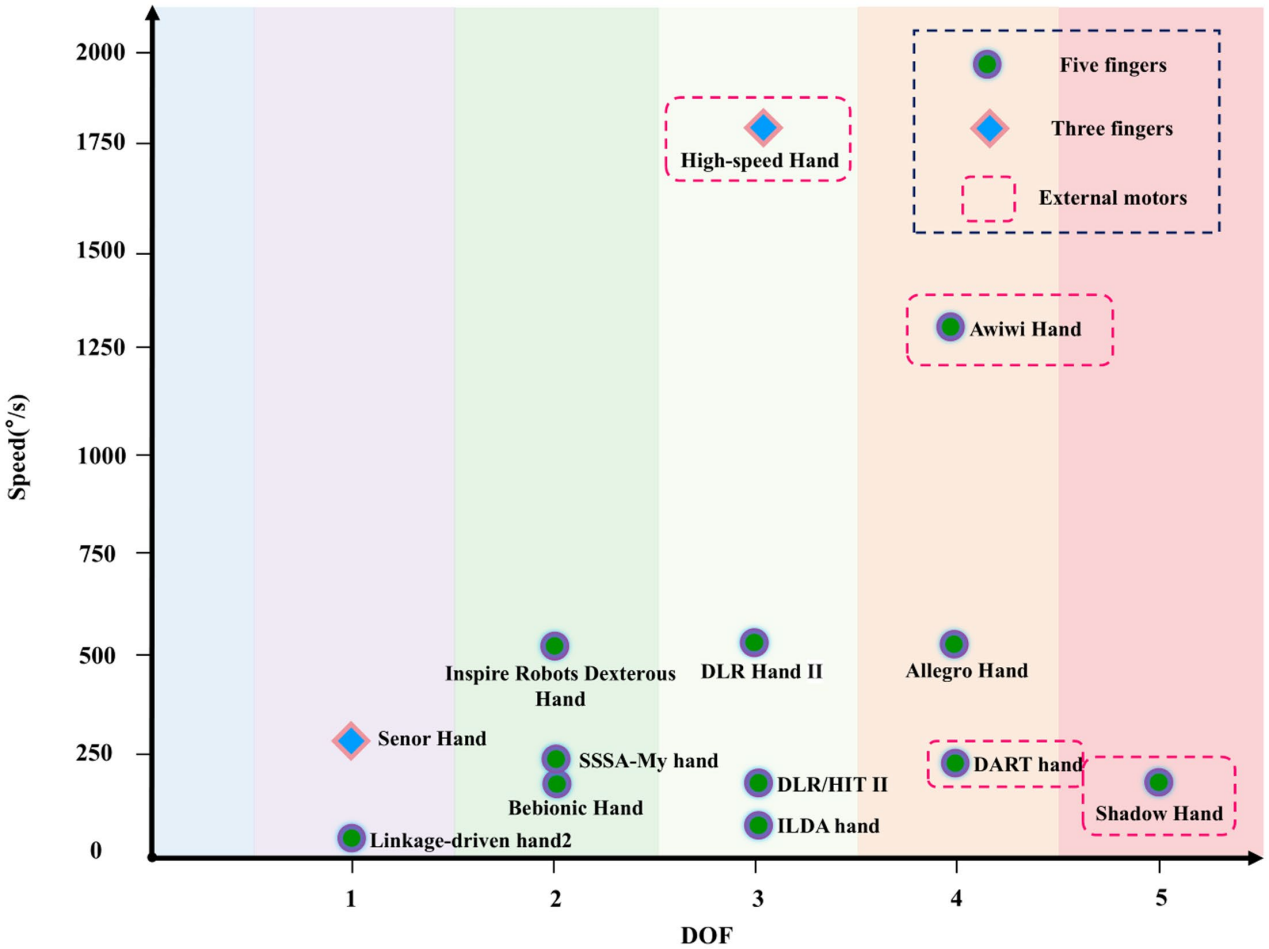
The DOF and speed distribution of different dexterous hands.

The distribution of DOF and speed for different dexterous hands is shown in Figure 4. Dexterous hand designs typically ensure certain grasping and manipulation capabilities, so the DOF is usually not very low. However, as DOF increases, the number of motors and speed requirements also rise, demanding higher motor performance, which becomes difficult to achieve within the limited space for actuation. Statistical results show that dexterous hands with a DOF of 1 or 5 are slower, while those with a DOF of 3 are the fastest, followed by DOFs 2 and 4. Hands with a DOF greater than 3 typically use external motors.

Response speed is critical for the dexterous hand, as it directly impacts the system’s sensitivity and accuracy. Fast response enhances the dexterous hand’s efficiency in performing complex tasks, improving both the accuracy and stability of operations, particularly in scenarios requiring fine control and rapid adjustments, such as robotic surgery, precision assembly, and human-computer interaction.

When designing high-speed dexterous hands, it is crucial not only to achieve fast motion but also to ensure sufficient fingertip force, which places high power demands on the actuators. However, a trade-off exists between actuator power and volume, necessitating careful control of actuator size. To address this challenge, many designs externalize the actuators to the forearm, employing tendon or belt transmission mechanisms for remote actuation. This approach helps optimize space and reduce the weight of the hand.

For instance, the RoboRay hand adopts this strategy, with motors externalized and tendon-driven transmission used to achieve a lightweight yet high-performance design. By minimizing the reduction ratio of the actuators, RoboRay hand achieves a maximum speed of 800°/s while maintaining 15 DOF. This design also improves system efficiency and response speed.

However, remote actuation methods, such as tendon transmission, introduce friction-related challenges. Friction can impair transmission accuracy, negatively impacting the precise control of joint positions in dexterous hands. Therefore, it is vital to consider the effects of friction on system performance during the design process and implement strategies to minimize its adverse impact.

### 4.2 Weight

In scenarios requiring high dynamic performance, such as piano playing, the weight of the dexterous hand is a critical factor to consider. On one hand, excessive weight can degrade the dynamic performance of the robotic arm, or even make it unable to bear the required load. On the other hand, a heavy dexterous hand can significantly affect the high dynamic performance of the fingers, limiting their ability to perform agile movements.

The weight of the human hand is about 0.4 kg (Tözeren, 1999), and the weight of the hand plus the forearm is about 1.5 kg. Existing dexterous hands are generally heavy, with most hands that have built-in motors weighing over 1 kg (Belter et al., 2013), while those with external motors often exceed 3 kg.

Existing humanoid dexterous hands with high DOF are typically much heavier than human hands. This excess weight places greater demands on the motion performance of humanoid robotic arms, affecting their flexibility and response speed.

Correlation analysis shows that the weight of the dexterous hand is most strongly correlated with DOF, followed by the driving form and the first-level transmission mode, with a relatively weak correlation to the structural form.

**Figure 5:**
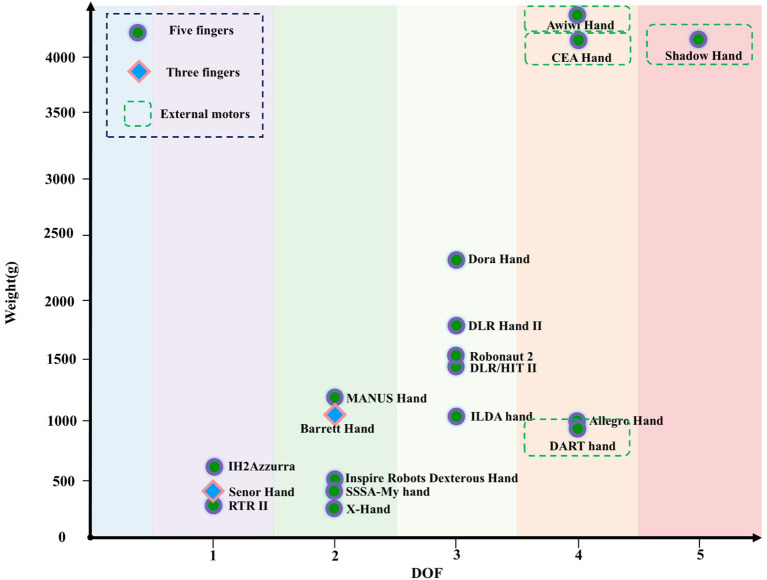
The DOF and weight distribution of different dexterous hands.

The DOF and weight distribution of different dexterous hands are shown in Figure 5. As the DOF increases, the number of motors increases. Dexterous hands with DOF greater than 3 mostly use external motors to increase the overall weight. Figure 5. shows that with the increase of DOF, the weight of dexterous hand gradually increases, showing a positive correlation.

The statistical results show that fully actuated dexterous hands are heavier than underactuated ones, because fully actuated dexterous hands require more motors, and almost all existing fully actuated dexterous hands weigh more than 0.5 kg.

In the first-level transmission mode, tendon-driven dexterous hands are heavier than linkage-driven ones. Tendon-driven mechanisms effectively transfer driving force, enabling complex motions and providing greater flexibility for dexterous hands.

Contrary to traditional assumptions, the correlation between the structural form and weight of dexterous hand is not strong. This is because, although dexterous hands with built-in motors are more common than in human hands, those with external motors tend to be heavier, resulting in a relatively low correlation between structural form and weight.

The main contributors to the weight of dexterous hands are the actuators and structural components. To reduce the weight of a humanoid dexterous hand closer to that of a human hand, efforts should focus on optimizing the materials used in the hand’s structure, using lighter and more efficient materials. Additionally, eliminating motors for unnecessary DOF is a highly effective strategy for significantly reducing overall weight, such as the Inspire Robots Dexterous Hand.

### 4.3 Fingertip force

Daily activities such as typing require fingers to move flexibly and gently, while in cases such as opening doorknobs or unscrewing bottle caps (Belter et al., 2013) fingers need to be held slowly and firmly. In factories and other environments, handling heavy or hard objects requires greater fingertip strength to ensure grasping ability and handling stability.

The fingertip force of the human hand typically ranges from 10 N to 15 N. In contrast, the fingertip force of most existing humanoid dexterous hands falls within the range of 10–40 N. Some advanced dexterous hands are capable of generating a maximum fingertip force of up to 100 N, providing a stronger gripping capability than the human hand.

**Figure 6:**
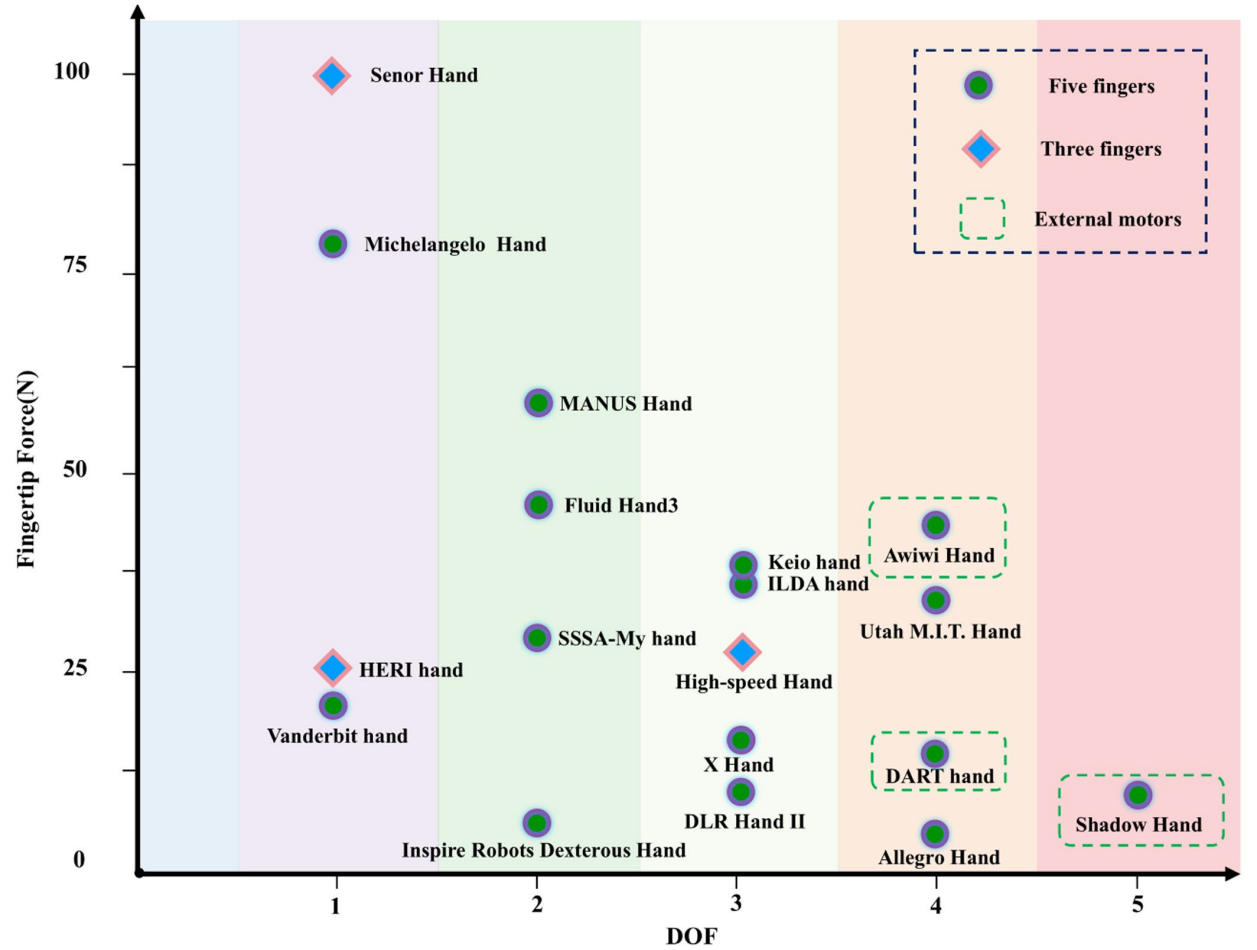
The DOF and fingertip force distribution of different dexterous hands.

The correlation between fingertip force and design variables is weak, but dexterous hands with higher fingertip forces tend to have fewer DOF. The relationship between the DOF of the dexterous hand and the fingertip force is shown in Figure 6. Dexterous hands with higher fingertip force require stronger mechanical structures to withstand greater forces, which increases weight and volume, limiting the achievable DOF.

Contrary to traditional understanding, the correlation between the structural form of the dexterous hand and fingertip force is relatively weak. This is because high fingertip force, which is typically required for handling tasks, does not necessarily require multiple DOF or specific motor configurations, whether built-in or external. In practice, dexterous hands with high fingertip force rely more on strong actuation and stable grasping than on the flexibility offered by high DOF.

In terms of fingertip force, the performance of existing dexterous hands has reached or even surpassed that of human hands. This is primarily achieved by sacrificing speed and DOF in favor of greater power and higher reduction ratios in the actuators, which increases output torque. For example, the Senor Hand reduces the number of DOF and uses more powerful motors, enabling it to generate fingertip forces of up to 100 N.

### 4.4 Compactness

In narrow operating environments such as industrial automation, service robots, and medical surgery, compact dexterous hands can work more efficiently and improve the efficiency and accuracy of task completion. The goal is to develop dexterous hands as compact as human fingers, avoiding any prominent features to adapt to narrow operating environments.

The compactness of human fingers, defined as the ratio of finger length to width, typically ranges from 5.3 to 5.6. In contrast, statistical data show that the compactness of most existing humanoid dexterous hand fingers ranges from 4 to 5.5, resulting in bulkier fingers compared to those of the human hand. This difference is closely linked to the complexity of the internal systems within dexterous hand fingers. Many dexterous hands incorporate a variety of sensors, such as tactile, temperature, and force sensors. Additionally, the driving mechanisms vary, including linkage-driven, tendon-driven, and gear-driven systems. Each of these systems occupies space in different ways, affecting overall space efficiency. While tendon-driven systems tend to occupy less volume than linkage-driven or gear-driven systems, the fixed components of tendon-driven designs often require more space.

The correlation between dexterous hand compactness and design variables is weak. As more data becomes available, the correlation between design variables and compactness may become clearer in the future.

To improve the compactness of dexterous hands, it is essential to eliminate unnecessary sensors and optimize the spatial arrangement of the driving form.

## 5 Coupling of performance metrics

The coupling of performance metrics is a critical aspect of dexterous hand design. In this section, we focus on exploring the relationships between two pairs of performance factors that are easier to measure: fingertip force and weight, and speed and fingertip force. These relationships are more clearly defined and offer valuable insights for optimizing the design of dexterous hands.

### 5.1 Fingertip force and weight

**Figure 7:**
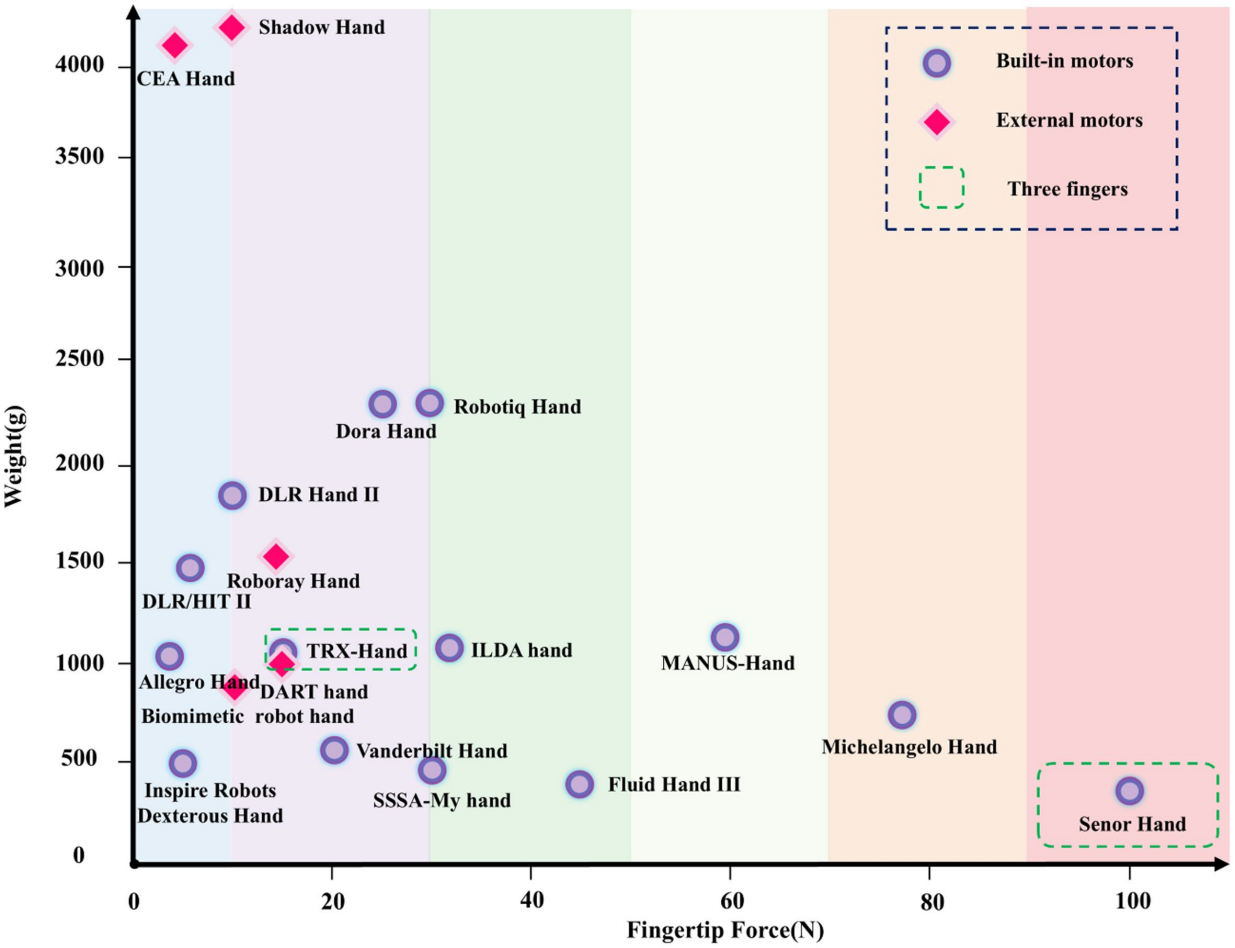
The fingertip force and weight distribution of different dexterous hands.

Contrary to traditional cognitive assumptions, fingertip force is negatively correlated with weight. As shown in Figure 7, dexterous hands with higher fingertip forces typically have lower DOF, which reduces the number of motors and simplifies the structure, thereby decreasing the overall weight. This design method helps minimize the structural complexity and energy losses associated with high DOF systems. For example, hands like the Senor Hand and MANUS Hand, which exhibit high fingertip force, feature very few DOF, with few motors that are integrated within the palm, resulting in a lightweight structure. In contrast, hands with higher DOF, such as the Shadow Hand, often have external motors and a larger overall weight, while their fingertip force is typically lower.

### 5.2 Speed and fingertip force

**Figure 8:**
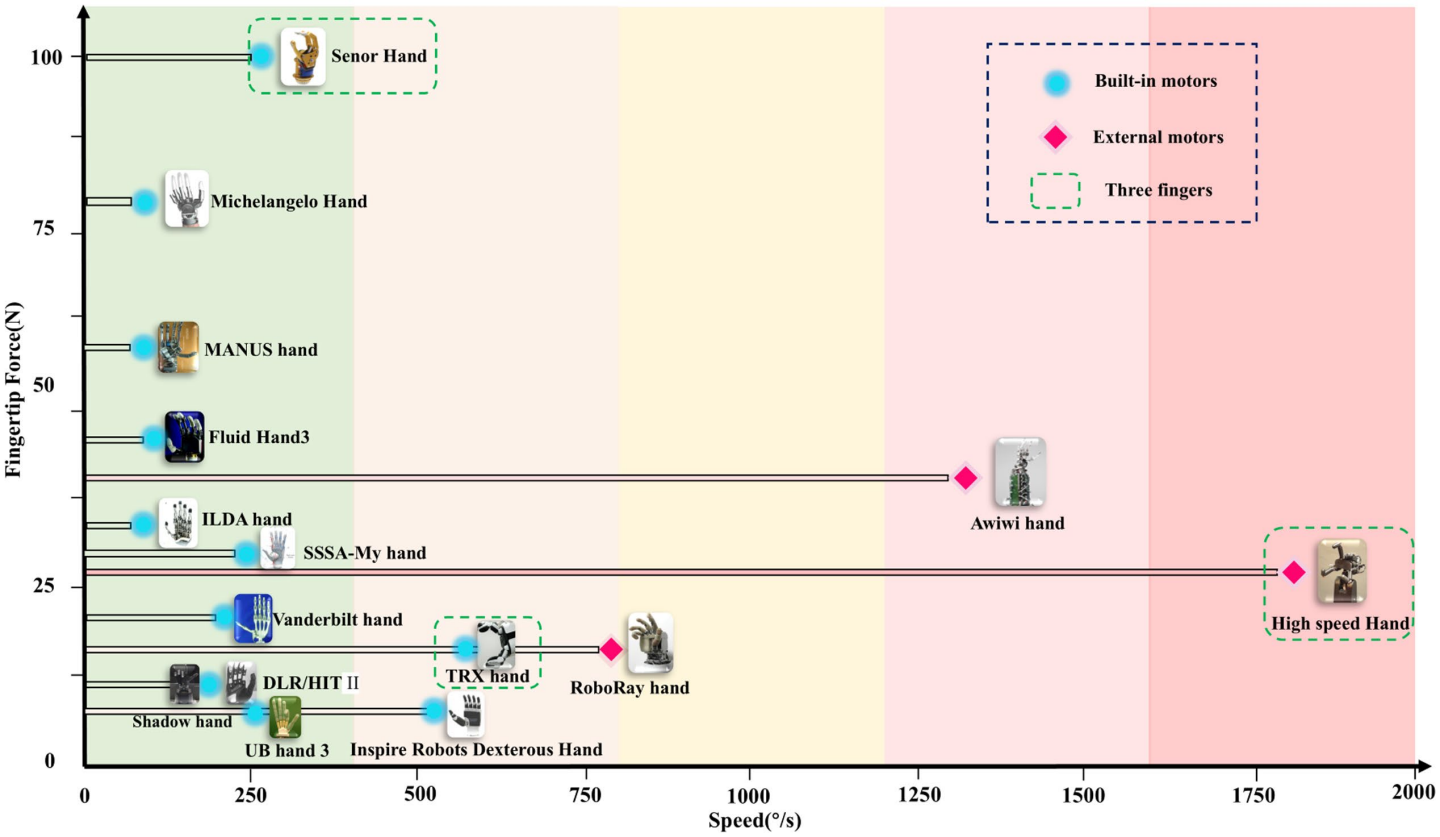
The speed and fingertip force distribution of different dexterous hands.

Due to power and heat dissipation limitations, dexterous hands face certain challenges in pursuing fingertip force and speed performance (Mohammadi et al., 2020), as shown in Figure 8. The fingertip force is mainly limited by the driving force. The greater the driving force, the higher the fingertip force, but at the same time, the power output and heat dissipation capacity of the motor need to be considered (Carrozza et al., 2004). A higher driving force can enhance the stability and strength of the dexterous hand during grasping and manipulation, but it may sacrifice speed response, as higher power output typically requires more complex heat dissipation, potentially limiting speed during operation (Pott et al., 2021), which may lead to limited speed when the dexterous hand is operating. Therefore, in the design of dexterous hand, it is necessary to comprehensively consider the balance between driving force, power output and heat dissipation capacity in order to achieve the best balance between fingertip force and speed performance.

**Figure 9:**
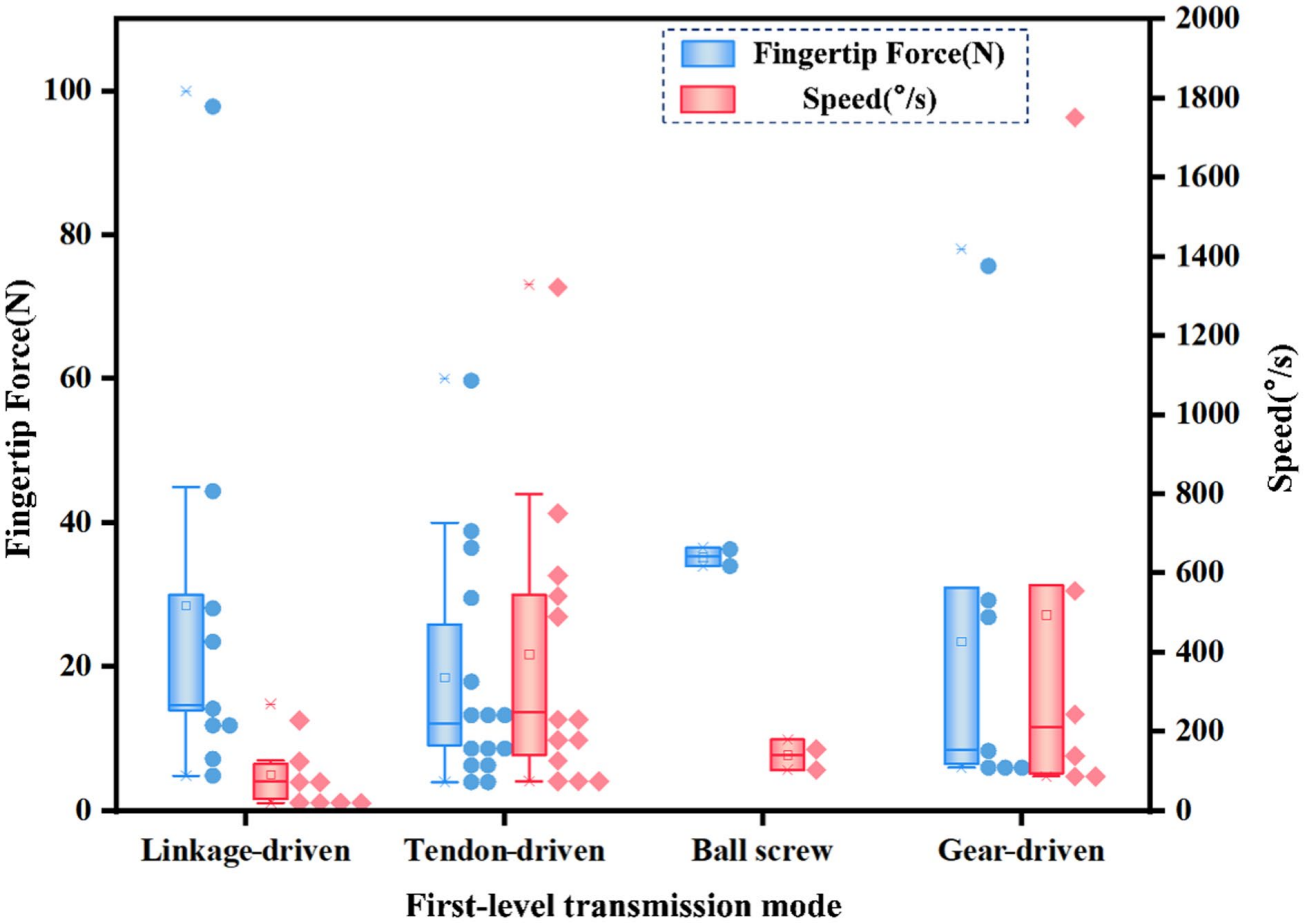
The first-level transmission mode and fingertip force and speed distribution of different dexterous hands.

In the first-level transmission mode, linkage-driven, tendon-driven, gear-driven, and ball screw-driven are usually used. The statistical first-level transmission mode and fingertip force and speed distribution are shown in Figure 9. The output fingertip force of the ball screw-driven is the highest and the speed is relatively low. The main reason is that the stroke of the ball screw-driven is small. Gear-driven and tendon-driven systems offer higher speed. If you want to balance the performance of fingertip force and speed, gear-driven systems seem to be the best method.

**Figure 10:**
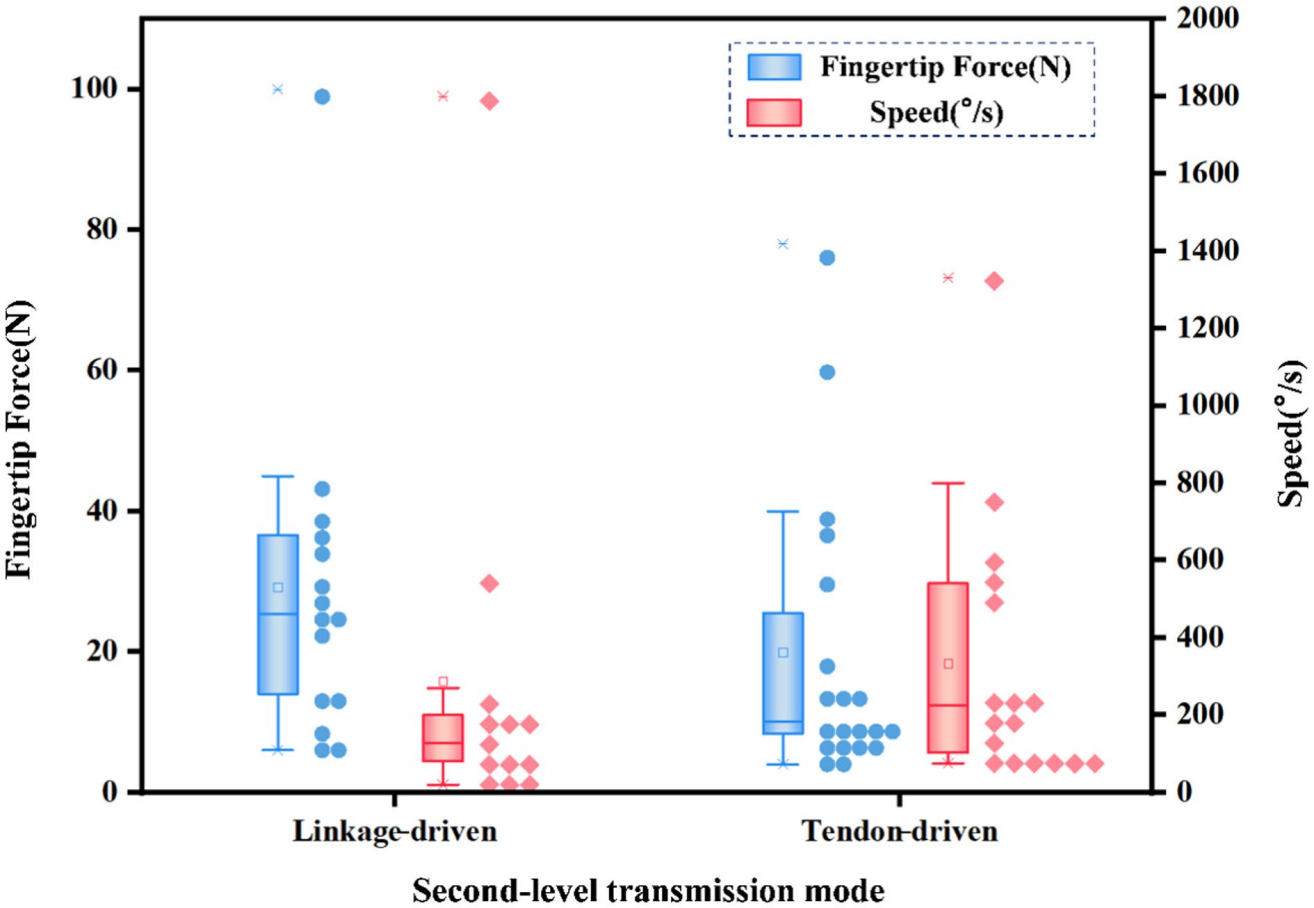
The second-level transmission mode and fingertip force and speed distribution of different dexterous hands.

In the second-level transmission mode, linkage-driven, tendon-driven, gear-driven, and belt-driven systems are typically used, though gear-driven and belt-driven systems are less common. The linkage-driven and tendon-driven are mainly counted, and the second-level transmission mode and fingertip force and speed distribution are shown in Figure 10. The linkage-driven system provides a higher output force but lower speed. The tendon-driven system has a lower output force but higher speed.

## 6 Discussion

In the context of dexterous hand applications, existing designs fall short of fully replicating the overall performance of the human hand. Thus, efforts should be directed at improving specific aspects of dexterous hands to optimize their functionality across various application scenarios.

To enable objective comparisons of dexterous hand performance metrics and offer clearer guidance for future research, this study introduces a standardized framework for performance evaluation. Five key criteria for performance evaluation are identified. These criteria include the following: DOF Ratio, Fingertip Force Ratio, Weight Ratio, Speed Ratio, and Compactness Ratio. These criteria were carefully selected because they align closely with the diverse requirements of various application scenarios, and they are easily quantifiable for comparison.

Each application scenario presents distinct performance needs for dexterous hands. For instance, in medical and assistive robotics, where flexibility and precise manipulation are critical, the number of DOF becomes a key consideration. In factory settings, the ability to exert strong grasping force is paramount, emphasizing the importance of fingertip force. In more dynamic and constrained environments, such as those requiring rapid movements or compact designs, speed and compactness become the primary priorities.

These five criteria are as follows:

1. DOF Ratio: The ratio of the DOFs of a dexterous hand to that of the human hand, with the human hand considered to have 21 DOFs.
2. Fingertip Force Ratio: The ratio of the fingertip force of a dexterous hand to that of a human hand, with the human fingertip force assumed to be 12 N.
3. Weight Ratio: The ratio of the weight of the dexterous hand to that of the human hand, with the weight of the human hand set to 0.5 kg. When considering the forearm, the weight ratio is compared to 1.5 kg for the human hand.
4. Speed Ratio: The ratio of the maximum speed of a dexterous hand to the maximum speed of a human hand, with the human hand’s maximum speed set to 2,290°/s.
5. Compactness Ratio: The ratio of the compactness of a dexterous hand to that of the human hand, with the human hand’s compactness set to 5.5.

**Figure 11:**
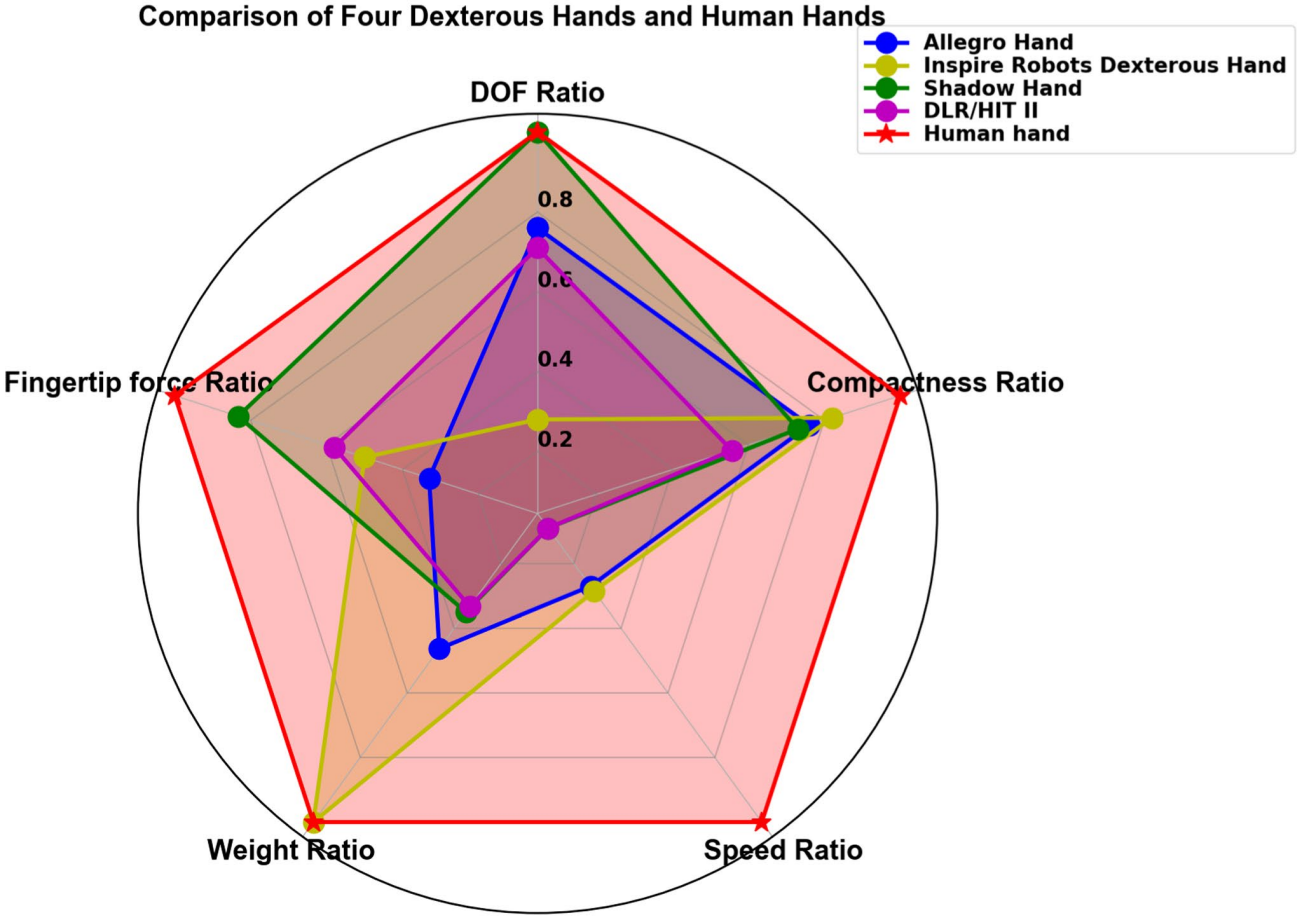
Comparison of four typical dexterous hands with a human hand.

By evaluating these five performance criteria, we can quantitatively compare various dexterous hands to the human hand, offering clear insights for improving dexterous hand designs. To further evaluate the relevance and effectiveness of these criteria, we apply them to four representative dexterous hands and compare their performance to that of the human hand, as shown in Figure 11. The Shadow Hand excels in terms of DOF and fingertip force, offering the greatest flexibility. This makes it especially advantageous for tasks involving the manipulation of objects in the palm. The Inspire Robots Dexterous Hand excels in speed and weight, making it highly suitable for tasks requiring high dynamics, such as playing the piano. The remaining two dexterous hands do not exhibit outstanding performance.

This standardized evaluation framework not only enables meaningful comparisons across different dexterous hands but also provides valuable guidance for future research and development in this field.

## 7 Conclusion

This paper focuses exclusively on the mechanical properties of dexterous hands, excluding aspects such as sensing, control, and electronics. By employing a data-driven statistical approach and using Cramér’s V, we quantified the correlation between different design variables and the performance of dexterous hands. Through the analysis of 65 existing models, we found that design variables such as DOF, structural form, driving form, and transmission mode significantly impact performance metrics like speed, weight, fingertip force, and compactness.

Based on these findings, we propose several design strategies to optimize dexterous hand performance across various application scenarios. For instance, to design high-speed dexterous hands, it is common to externalize the motors to the forearm, using tendon or belt transmission mechanisms to minimize reduction ratios in gearboxes, or even to adopt direct-drive systems. To reduce the weight of humanoid dexterous hands, efforts should focus on optimizing the materials used in the hand’s structure and eliminating motors associated with unnecessary DOF. To enhance fingertip force, it is possible to sacrifice speed and DOF, employing more powerful motors and higher reduction ratios in the gearboxes. To improve the compactness of dexterous hands, removing unnecessary sensors and optimizing the spatial arrangement of the driving form is essential.

The proposed performance evaluation criteria can be used to compare the performance of different dexterous hands with that of the human hand, providing clear guidance for future design optimizations. While not all mechanical design challenges discussed in this study have been fully resolved, this paper lays a critical foundation for designers working to enhance the functionality of dexterous hands.

In the future, the development of dexterous hands may have the following directions:

1. Enhancing robustness: As one of the most exposed and fragile components of a robotic system, the dexterous hand is costly to produce. To ensure that the dexterous hand can perform tasks stably and reliably in complex, dynamic, and uncertain environments, methods such as energy storage systems could be explored to enhance its robustness. This would help the dexterous hand maintain functionality in the face of unexpected situations or external disturbances.
2. Improving grasping adaptability: Currently, most dexterous fingertips use rigid materials to enhance accuracy, but their adaptability in grasping is limited. In the future, the use of local flexibility could improve the adaptability of the dexterous hand, enabling it to better adjust to changes in the position of the object being grasped, thus reducing the need for constant replanning. This will allow the dexterous hand to perform more effectively across a wide range of tasks.
3. Multi-finger design: With advances in control technology, sensor technology, and materials science, dexterous hands are no longer restricted to traditional five-finger designs. The possibility of six-finger or even more-finger designs should be considered to provide greater degrees of freedom and operational capability. Such a design would allow the dexterous hand to perform more complex and detailed tasks, further expanding its potential applications.
