# Supplementary material for: Critical review on the relationship between design variables and performance of dexterous hands: a quantitative analysis
Source: Front Neurorobot. 2025 Jan 30;18:1513458. doi: 10.3389/fnbot.2024.1513458 (PMC11821616; doi:10.3389/fnbot.2024.1513458)
Supplement: Supplementary file 1 [file Data_Sheet_1.PDF]

Supplementary Material

Table 1

Detailed statistical data of dexterous hand.

	Transmission mode	DOF	Driving form	Structural form	Fingertip force(N)	Weight(Kg)	Speed(°/s)	Compactness	Time
1. ILDA hand[12]	Ball screw-driven and linkage-driven	3	Underactuated	Built-in	34	1.1	81	3.27	2021
2. Inspire Robots Dexterous Hand[13]	Linkage-driven	2	Underactuated	Built-in	6	0.5	570	4.5	2019
3. Shadow Hand[14]	Tendon-driven	5	Fully actuated	External	10	4.3	200	4.04	2004
4. SSSA-My hand[15]	Gear-driven and tendon-driven	2	Underactuated	Built-in	31	0.48	250	-	2016
5. DLR/HIT II[16]	Gear-driven and linkage-driven	3	Fully actuated	Built-in	7	1.5	180	3.06	2008
6. Robotiq Hand[17]	Linkage-driven	1	Underactuated	Built-in	30	2.3	-	-	2014
7. Bebionic hand [18]	Ball screw-driven and linkage-driven	2	Underactuated	Built-in	36.6	-	180	-	2011
8. Four-finger tendon-driven hand[19]	Tendon-driven and linkage-driven	3	Underactuated	Built-in	-	0.5	-	6.1	2017
9. Biomimetic robot hand[20]	Tendon-driven	3	Underactuated	External	10	0.942	80	7.52	2016
10. MCR-Hand II[21]	Linkage-driven	4	Fully actuated	Built-in	3	0.8	-	-	2021
11. DART hand[22]	Tendon-driven	4	Fully actuated	External	14.4	1.05	250	-	2011
12. DLR Hand II[23]	Tendon-driven	3	Underactuated	Built-in	10	1.8	540	-	2003
13. Awiwi hand[24]	Tendon-driven	4	Fully actuated	External	40	4.5	1330	-	2014
14. X-hand [25]	Tendon-driven	2	Underactuated	Built-in	8.3	0.28	142	-	2022
15. IH2Azzurra[26]	Tendon-driven	1	Underactuated	Built-in	-	0.64	-	-	2023
16. Allegro Hand[27]	Tendon-driven	4	Fully actuated	Built-in	4	1.08	545	4.19	-
17. TRX-Hand[28]	Tendon-driven	-	-	Built-in	15	1.16	600	-	2023
18. Fluid Hand3[29]	-	2	Underactuated	Built-in	45	0.4	90	-	2009
19. Vanderbilt hand [30]	Tendon-driven	1	Underactuated	Built-in	20	0.58	225	4.81	2009
20. UB hand 3[31]	Tendon-driven	4	Fully actuated	External	6.8	-	250	4.9	2005
21.Senor Hand[32]	Linkage-driven	1	Underactuated	Built-in	100	0.4	270	-	-
22. MANUS hand[33]	Tendon-driven	2	Underactuated	Built-in	60	1.2	80	-	2004
23. Gifu hand II[34]	Gear-driven and linkage-driven	4	Fully actuated	Built-in	4.9	-	-	-	1999

Continuation Table 1

Some statistical data of dexterous hand.

	Transmission mode	DOF	Driving form	Structural form	Fingertip force(N)	Weight(Kg)	Speed(°/s)	Compactness	Time
24. Linkage-driven hand1[35]	Linkage-driven	2	Underactuated	Built-in	10	0.5	-	-	2009
25. Vincent Hand[36]	Gear-driven and tendon-driven	2	Underactuated	Built-in	8.44	-	103	-	-
26. iLimb[37]	Gear-driven and tendon-driven	2	Underactuated	Built-in	6.54	0.615	95	5.53	2009
27. Linkage-driven hand2[38]	Linkage-driven	1	Underactuated	External	14	0.28	20	-	2001
28. Linkage-driven hand3[39]	Linkage-driven	2	Underactuated	Built-in	45	-	80	4.97	2001
29. Linkage-driven hand4[40]	Linkage-driven	2	Underactuated	Built-in	14.7	-	126.76	5.62	2017
30. Dora Hand[41]	Linkage-driven	3	Fully actuated	Built-in	25	2.3	70	4.17	2022
31. Robonaut 2[42]	Tendon-driven and linkage-driven	3	Underactuated	External	22.5	1.59	-	5.7	2010
32. HERI Hand[43]	Tendon-driven and linkage-driven	1	Underactuated	Built-in	25.4	-	-	-	2017
33. JQ3[44]	-	3	Fully actuated	Built-in	20	0.95	90	4.8	-
34. High speed hand[45]	Gear-driven and linkage-driven	3	Fully actuated	External	28	＞1	1800	-	2003
35. Michelangelo hand[46]	Gear-driven and tendon-driven	1	Underactuated	Built-in	78	0.75	87		2012
36. Utah M.I.T. Hand[47]	Tendon-driven	4	Fully actuated	External	31.8	＞1	-	-	1983
37. X Hand[48]	-	3	Fully actuated	Built-in	16	-	-	-	-
38. CEA hand[49]	Tendon-driven	4	Fully actuated	External	4.2	4.2	-	-	2015
39. DEXMART hand[50]	Tendon-driven	3	Fully actuated	External	10	＞1	-	-	2014
40. Integrated robotic hand[51]	Tendon-driven	3	-	-	10	-	-	-	2014
41. RoboRay hand[52]	Tendon-driven	3	Fully actuated	External	15	1.59	800	-	2014
42. Barrett Hand[53]	Gear-driven and belt-driven	1	Underactuated	Built-in	20	1.18	-	-	1988
43. Self-adapting auxiliary hand[54]	Linkage-driven	3	Underactuated	External	40	5	-	5.41	-
44. Remedi Hand[55]	Linkage-driven	2	Underactuated	-	9.2	0.4	40	-	2000
45. Keio Hand[56]	Tendon-driven	3	Fully actuated	External	37	0.73	108	-	2008

Table 2

Partial statistical data of dexterous hand.

	Transmission mode	DOF	Driving form	Structural form	Weight(Kg)	Compactness	Time
1. RTR II[57]	Tendon-driven	1	Underactuated	Built-in	0.35	-	2002
2. UNB Hand[58,59]	Linkage-driven	1	Underactuated	-	-	-	2010
3. Schunk 5-Finger Gripping Hand [60]	Ball screw-driven and linkage-driven	2	Underactuated	Built-in	-	-	2014
4. Ritsumeikan robotic Hand[61]	Tendon-driven	2	Underactuated	Built-in	0.4	4.1	2013
5. An underactuated anthropomorphic hand[62]	Tendon-driven	3	Underactuated	External	-	-	2014
6. LISA Hand[63]	Linkage-driven	3	Underactuated	Built-in	-	2.79	2012
7. Linkage-driven hand5[64]	Linkage-driven	2	Underactuated	Built-in	-	-	2001
8. Linkage-driven hand6[65]	Linkage-driven	2	Underactuated	Built-in	-	-	2017
9. Linkage-driven hand7[66]	Linkage-driven	2	Underactuated	Built-in	-	-	2000
10. Linkage-driven hand8[67]	Linkage-driven	3	Underactuated	Built-in	-	-	2016
11. Linkage-driven hand9[68]	Linkage-driven	3	Underactuated	-	-	-	2012
12. Linkage-driven hand10[69]	Linkage-driven	3	Underactuated	Built-in	-	3.68	2009
013. Optimus hand[70]	Tendon-driven	1	Underactuated	Built-in	-		-
14. iCub hand[71]	Tendon-driven	2	Underactuated	External	-	5.6	2010
15. Skill Hand[72]	-	3	Underactuated	Built-in	-	-	-
16. Linkage-driven hand11[73]	Linkage-driven	3	Fully actuated	Built-in	-	-	2021
17. COSA-GRS hand[74]	Linkage-driven	2	Underactuated	Built-in	-	4.76	2013
18. UA hand[75]	Linkage-driven	2	Underactuated	-	-	3.46	2017
19. Linkage-driven hand12[76]	Linkage-driven	3	Fully actuated	-	-	6.23	2007
20. Linkage-driven hand13[77]	Linkage-driven	1	Underactuated	-	0.25	3.8	2017

Table3 Distribution of Fingertip Force by DOF Category

	Fingertip force < 12	Fingertip force ≥ 12	Row Total
DOF < 3	6	13	19
DOF ≥ 3	11	12	23
Column Total	17	25	42

Table4 Distribution of Weight by DOF Category

	Weight < 0.5	Weight ≥ 0.5	Row Total
DOF < 3	9	9	18
DOF ≥ 3	0	20	20
Column Total	9	29	38

Table5 Distribution of Speed by DOF Category

	Speed < 200	Speed ≥ 200	Row Total
DOF < 3	11	4	15
DOF ≥ 3	6	8	14
Column Total	17	12	29

Table6 Distribution of Compactness by DOF Category

	Compactness < 5.5	Compactness ≥ 5.5	Row Total
DOF < 3	3	2	5
DOF ≥ 3	8	3	11
Column Total	11	5	16

Table7 Distribution of Fingertip Force by Driving Form Category

	Fingertip force < 12	Fingertip force ≥ 12	Row Total
Fully actuated	8	9	17
Underactuated	8	17	25
Column Total	16	26	42

Table8 Distribution of Weight by Driving Form Category

	Weight < 0.5	Weight ≥ 0.5	Row Total
Fully actuated	0	14	14
Underactuated	9	14	23
Column Total	9	28	37

Table9 Distribution of Speed by Driving Form Category

	Speed < 200	Speed ≥ 200	Row Total
Fully actuated	4	7	11
Underactuated	15	3	18
Column Total	19	10	29

Table10 Distribution of Compactness by Driving Form Category

	Compactness < 5.5	Compactness ≥ 5.5	Row Total
Fully actuated	7	1	8
Underactuated	11	7	18
Column Total	18	8	26

Table11 Distribution of Fingertip force by Structural Form Category

	Fingertip force < 12	Fingertip force ≥ 12	Row Total
External	3	8	11
Built-in	11	20	31
Column Total	14	28	42

Table12 Distribution of Weight by Structural Form Category

	Weight < 0.5	Weight ≥ 0.5	Row Total
External	0	8	8
Built-in	8	21	29
Column Total	8	29	37

Table13 Distribution of Speed by Structural Form Category

	Speed < 200	Speed ≥ 200	Row Total
External	2	5	7
Built-in	16	7	23
Column Total	18	12	30

Table14 Distribution of Compactness by Structural Form Category

	Compactness < 5.5	Compactness ≥ 5.5	Row Total
External	4	2	6
Built-in	12	5	17
Column Total	16	7	23

Table15 Distribution of Fingertip force by the First-level Transmission Mode Category

	Fingertip force < 12	Fingertip force ≥ 12	Row Total
Linkage-driven	3	7	10
Tendon-driven	9	10	19
Column Total	12	17	29

Table16 Distribution of Weight by the First-level Transmission Mode Category

	Weight < 0.5	Weight ≥ 0.5	Row Total
Linkage-driven	4	4	8
Tendon-driven	3	17	20
Column Total	7	21	28

Table17 Distribution of Speed by the First-level Transmission Mode Category

	Speed < 200	Speed ≥ 200	Row Total
Linkage-driven	5	1	6
Tendon-driven	4	9	13
Column Total	9	10	19

Table18 Distribution of Compactness by the First-level Transmission Mode Category

	Compactness < 5.5	Compactness ≥ 5.5	Row Total
Linkage-driven	8	1	9
Tendon-driven	4	4	8
Column Total	12	5	17

Table19 Distribution of Fingertip force by the Second-level Transmission Mode Category

	Fingertip force < 12	Fingertip force ≥ 12	Row Total
Linkage-driven	6	12	18
Tendon-driven	12	7	19
Column Total	18	19	37

Table20 Distribution of Weight by the Second-level Transmission Mode Category

	Weight < 0.5	Weight ≥ 0.5	Row Total
Linkage-driven	4	11	15
Tendon-driven	4	16	20
Column Total	8	27	35

Table21 Distribution of Speed by the Second-level Transmission Mode Category

	Speed < 200	Speed ≥ 200	Row Total
Linkage-driven	6	6	12
Tendon-driven	7	9	16
Column Total	13	15	28

Table22 Distribution of Compactness by the Second-level Transmission Mode Category

	Compactness < 5.5	Compactness ≥ 5.5	Row Total
Linkage-driven	11	3	14
Tendon-driven	5	3	8
Column Total	16	6	22
